# p53-Dependent PUMA to DRAM antagonistic interplay as a key molecular switch in cell-fate decision in normal/high glucose conditions

**DOI:** 10.1186/s13046-017-0596-z

**Published:** 2017-09-11

**Authors:** Alessia Garufi, Giuseppa Pistritto, Silvia Baldari, Gabriele Toietta, Mara Cirone, Gabriella D’Orazi

**Affiliations:** 1 Department of Research, Advanced Diagnostics, and Technological Innovation, Translational Research Area, Regina Elena National Cancer Institute, Rome, Italy; 2 Department of Medical, Oral and Biotechnological Sciences, Tumor Biology Section, University “G. d’Annunzio”, Via de Vestini, 31, 66013 Chieti, Italy; 3 Department of Systems Medicine, University “Tor Vergata”, Rome, Italy; 4 Department of Experimental Medicine, Institute Pasteur Cenci Bolognetti Foundation, Sapienza University, Rome, Italy

**Keywords:** p53, DRAM, PUMA, Autophagy, Hyperglycemia, Diabetes, Chemotherapy, Cancer

## Abstract

**Background:**

As an important cellular stress sensor phosphoprotein p53 can trigger cell cycle arrest and apoptosis and regulate autophagy. The p53 activity mainly depends on its transactivating function, however, how p53 can select one or another biological outcome is still a matter of profound studies. Our previous findings indicate that switching cancer cells in high glucose (HG) impairs p53 apoptotic function and the transcription of target gene PUMA.

**Methods and results:**

Here we report that, in response to drug adriamycin (ADR) in HG, p53 efficiently induced the expression of DRAM (damage-regulated autophagy modulator), a p53 target gene and a stress-induced regulator of autophagy. We found that ADR treatment of cancer cells in HG increased autophagy, as displayed by greater LC3II accumulation and p62 degradation compared to ADR-treated cells in low glucose. The increased autophagy in HG was in part dependent on p53-induced DRAM; indeed DRAM knockdown with specific siRNA reversed the expression of the autophagic markers in HG. A similar outcome was achieved by inhibiting p53 transcriptional activity with pifithrin-α. DRAM knockdown restored the ADR-induced cell death in HG to the levels obtained in low glucose. A similar outcome was achieved by inhibition of autophagy with cloroquine (CQ) or with silencing of autophagy gene ATG5. DRAM knockdown or inhibition of autophagy were both able to re-induce PUMA transcription in response to ADR, underlining a reciprocal interplay between PUMA to DRAM to unbalance p53 apoptotic activity in HG. Xenograft tumors transplanted in normoglycemic mice displayed growth delay after ADR treatment compared to those transplanted in diabetics mice and such different in vivo response correlated with PUMA to DRAM gene expression.

**Conclusions:**

Altogether, these findings suggest that in normal/high glucose condition a mutual unbalance between p53-dependent apoptosis (PUMA) and autophagy (DRAM) gene occurred, modifying the ADR-induced cancer cell death in HG both in vitro and in vivo.

## Background

In response to several types of genotoxic stress the p53 oncosuppressor is activated to control, as transcription factor, genes regulating different cellular outcomes such as cell-cycle arrest and apoptosis [1]. In this manner, p53 protects cells from genomic instability leading to tumorigenesis, reduces tumor progression, and activates the apoptotic response of tumor cells to anticancer drugs [2]. Given its key role in restraining tumorigenesis and tumor progression, p53 is frequently mutated in over 50% of human cancer types and indirectly inactivated in the other 50%, indicating that the presence of a functional p53 pathway is incompatible with neoplastic cell growth [3]. One of the most dramatic effects of p53 activation is the apoptotic clearance of cancer cells [4] which is one of the two ideal goals of anticancer therapy, the other being the stimulation of host tumor-specific response, both cooperating in the achievement of clinically relevant effects [5, 6]. Apoptotic signals can engage two main pathways (the extrinsic and the intrinsic) which are interconnected [7]. P53 is involved in both pathways through the regulation of target genes that encode, for instance, the mitochondrial BH3-domain proteins NOXA and PUMA [8, 9]. PUMA (p53 upregulated modulator of apoptosis) represents one of the most potent pro-apoptotic BH3-only proteins and a key mediator of p53-dependent apoptosis in response to a wide variety of stress signals including genotoxic stress, double- and single-stranded DNA breaks (i.e., UV, γ-IR, purine analogues, topoisomerase inhibitors, chemotherapeutic agents, etc.) but also oxidative stress, neurotoxins, changes in microtubule structure, deficiency of growth factors, hypoxia and viral infection [10]. The absence of PUMA has been shown to cause high resistance of cancer cells to apoptosis induced by DNA-damaging agents, such as adriamycin, 5-fluorouracil, cisplatin, etc. [11, 12].

Besides, p53 has been shown to play a critical role in p53 in regulation of autophagy, a catabolic pathway by which eukaryotic cells degrade and recycle macromolecules and organelles, particularly under conditions of nutrient deprivation [13]. This may depend on p53 subcellular localization and/or by p53 transcription-dependent and -independent activities [14–16]. TP53 may induce autophagy through, for instance, activation of AMPK kinase/mTOR signalling [17] or by transcriptional induction of autophagy genes such as DRAM (damage-regulated autophagy modulator), a lysosomal protein and a stress-induced regulator of autophagy [18]. DRAM has been shown to be not only critical for the ability of p53 to induce autophagy, but also for p53-induced apoptotic cell death [19], contributing to the complex mechanisms that regulate whether or not a cell dies in response to p53. Thus, although p53 is involved in the regulation of autophagy, the exact nature of this link remains seemingly controversial [20].

Autophagy, along with apoptosis controls the turnover of organelles, proteins and therefore the cell fate (survival/cell death). Generally autophagy blocks the induction of apoptosis that shuts off the autophagic process and the dialogue between these two pathways influences the normal clearance of dying cells [21]. Thus, autophagy inhibits apoptosis in mammalian cells [22] and facilitates the resistance of tumor cells to anticancer agents [23]. Therefore, knowing the mechanisms that rule the interplay between these two cellular processes (autophagy/apoptosis) has important pathophysiological consequences [24]. Recently we reported that high glucose (HG) condition reduces the tumor cell response to drug-induced apoptosis due to impairment of p53 apoptotic function. Mechanistically we found that HG induces homeodomain-interacting protein kinase 2 (HIPK2) protein degradation leading to impairment of HIPK2/p53 apoptotic axis with inhibition of p53 apoptotic targets PUMA and p53AIP1 [25, 26].

In this study, we present evidence that HG condition changes the p53 transcriptional activity from PUMA to DRAM with the consequence of impairment of drug-induced cell death. We also show that p53-induced DRAM sustains HG-triggered autophagy and that either DRAM or autophagy inhibition restore both PUMA transcription and drug-induced cell death in vitro and in vivo.

## Methods

### Cell culture and reagents

In this study colon cancer RKO, HCT116, and HCT116-p53^−/−^ cells were used. Cells were routinely cultured in DMEM (Dulbecco’s Modified Eagle’s medium) (Life Technology-Invitrogen, Eggenstein, Germany) containing 1 g/L D-glucose (low glucose - LG), supplemented with 10% heat-inactivated fetal bovine serum (FBS) plus 100 units/ml penicillin/streptomycin, and glutamine, in 5% CO_2_ humidified incubator at 37 °C. For high glucose (HG) condition, low glucose culture medium was replaced with DMEM containing 4.5 g/L D-glucose (Life Technology-Invitrogen) supplemented with 2% FBS for 24 h, as previously reported [25–28], before performing other treatments for the indicated times.

To generate a cell line stably expressing GFP-LC3, 3 × 10^5^ RKO cells were cultured into 6-well plates and transfected with 5 μg of pEGFP-LC3 expression vector (kindly provided by Moshe Oren, Weizmann Institute of Science, Rehovot, Israel) with Lipofectamine Plus (Invitrogen, Monza, Italy) according to the manufacturer’s instructions. Forty-eight hours after transfection cells underwent selection with geneticin G418 (1.5 mg/ml). After 10–14 days, the selected GFP-LC3-positive clones were visualized on a Nikon Eclipse Ti-U fluorescence microscope (Nikon).

Chemotherapeutic drug Adriamycin (ADR) (Sigma) was added in culture medium at 2 μg/ml for 16–24 h; the inhibitor of autophagic protein degradation chloroquine (CQ) [29] (Sigma-Aldrich) was added in culture medium at 25 μM for 16 h; p53 inhibitor pifithryn-α (PFT-α) [30] (ENZO Life Sciences, Lausen Switzerland) was used at 30 μM for 16–24 h, as reported [31].

### Viability assay

For viability assay, subconfluent cells were plated in duplicate in 60 mm multiwell Petri dishes and 24 h later culture medium was replaced with HG or low glucose medium, both containing 2% FBS. The day after, ADR (2 μg/ml) were added to cell cultures for 16–24 h. Both floating and adherent cells were collected and cell viability was determined by Trypan blue (Sigma, St. Louis, MO, USA) exclusion by direct counting with a haemocytometer. The percentage of cell death, as blue/total cells, was assayed by scoring about 200 cells per well in triplicate.

### Cell death/PI staining

Cell death was quantified by Fluorescence Activated Cell Sorting (FACS) analysis, staining cells with the nonvital dye propidium iodide (PI) (Immunological Sciences, Rome, Italy), following the manufacturer’s instruction [32]. Briefly, floating cells were collected by centrifugation and pooled with adherent cells recovered from the plates, fixed in 80% ethanol and stained in a PBS solution containing PI (62.5 mg/ml; Sigma-Aldrich), and RNase A (1.125 mg/ml; Sigma-Aldrich). Samples were acquired with a FACScan instrument (Becton Dickinson Europe Holdings SAS - Le Pont De Claix, France) and the percentage of cells in sub-G1 compartment was calculated using ModFit LT software (Becton Dickinson). About 30.000 events were acquired and gated using forward scatter and side scatter to exclude cell debris.

### Western blot analysis

Total cell extracts were prepared by incubation in lysis buffer (50 mM Tris-HCl, pH 7.5, 150 mM NaCl, 5 mM EDTA, pH 8.0, 150 mM KCl, 1 mM dithiothreitol, 1% Nonidet P-40) and a mix of protease and phosphatase inhibitors (Sigma-Aldrich). Protein concentration was then determined using BCA Protein Assay kit (Bio-Rad, Hercules, CA, USA). Samples were then denatured in SDS sample buffer. Total proteins were separated by loading 20–60 μg of total cell lysates on denaturing 6–20% SDS-PAGE (Bio-Rad) and transferred to polyvinylidene difluoride (PVDF) (Merck Millipore, Billerica, MA, USA) or nitrocellulose (Bio-Rad) membranes. Unspecific binding sites were blocked by incubating membranes for 1 h in 0.05% Tween-20 (*v*/v in TBS) supplemented with 5% non-fat powdered milk or bovine serum albumin, followed by overnight incubation with the following primary antibodies: rabbit polyclonal anti-LC3B (Sigma-Aldrich), mouse monoclonal anti-p62 (SQSTM1, D-3) (Santa Cruz Biotechnology, Dallas, TX, USA); mouse monoclonal anti-poly(ADP-ribose) polymerase (PARP, cleavage site-214/215, Millipore); and mouse monoclonal anti-β-actin (Calbiochem, San Diego, CA, USA). Primary antibodies were detected with appropriate anti-immunoglobulin-G-horseradish peroxidase secondary antibodies (Bio-Rad). Enzymatic signals were visualized using chemiluminescence (ECL Detection system, Amersham GE Healthcare, Milan, Italy). Images were acquired with the EPSON Expression 10,000 XL scanner (Epson, Long Beach, CA, USA) and densitometry was performed with the ImageJ software (NIH, Bethesda, MD, USA).

### RNA extraction and semi-quantitative reverse transcription (RT)-PCR analysis

Cells were harvested in TRIzol Reagent (Life Technology-Invitrogen) and total RNA was isolated following the manufacturer’s instructions, as previously reported [25]. The first strand cDNA was synthesized from 2 μg of total RNA with MuLV reverse transcriptase kit (Applied Biosystems, Foster City, CA, USA). Semi-quantitative Reverse-Transcribed (RT)-PCR was carried out by using Hot-Master Taq polymerase (Eppendorf, Milam Italy) with 2 μl cDNA reaction and genes specific oligonucleotides under conditions of linear amplification. PCR products were run on a 2% agarose gel and visualized with ethidium bromide. The housekeeping 28S gene, used as internal standard, was amplified from the same cDNA reaction mixture. Densitometric analysis was applied to quantify mRNA levels compared to control gene expression.

### siRNA interference

Cells were plated at semiconfluence in 35-mm Petri dishes the day before transfection. Control-siRNA, siDRAM (sc-96,209; Santa Cruz) and siATG5 (Dharmacon, Thermo Scientific, Milan, Italy) were transfected overnight using Lipofectamine Plus reagent (Invitrogen). DRAM silencing was evaluated 48 h after transfection by RT-PCR analysis.

### In vivo tumor growth

In vivo experiment was performed by using six-week-old CD-1 male nude (nu/nu) mice (Charles River Laboratories). They were housed in specific pathogen-free conditions and fed standard cow pellets and water ad libitum. Studies were performed in accordance with the National Cancer Institute Regina Elena standard guidelines for animal care; all experimental protocol were approved by the Ethical Committee for animal research of the National Cancer Institute Regina Elena in Rome, Italy, and by the Italian Ministry of Health, and performed in accordance with the Italian and European legislation. For diabetes induction the mice were injected intraperitoneally with a single high dose of 160 to 240 mg/kg streptozotocin (STZ) (Sigma-Aldrich) which is considered to achieve consistently a diabetic state with limited morbidity and mortality [33]. The mice were subsequently hydrated with 1.0 ml normal physiologic saline administrated subcutaneously. Blood was obtained by lancet prick in the tail. Blood glucose concentration was monitored once before and daily after STZ injection until a diabetic state was confirmed by the glucose dehydrogenase method. After mice reached a glucose concentration exceeding 300 mg/dl (considered diabetic), solid tumors were obtained by injecting i.m. on the flank of each mouse 4 × 10^6^ viable RKO cells suspended in 0.1 ml PBS. The mice were examined every day after injection until tumors reached approximately 300 mm^3^ volume (about 7 days from injection). Mice were then randomized in four groups (6–8 mice/group) and injected with ADR (10 mg/kg body weight) i.p or PBS as control, as follow: 1) normoglycemic/PBS (Mock), 2) normoglycemic/ADR, 3) diabetic/PBS, 4) diabetic/ADR. Tumor dimensions were measured every other day and their volumes were calculated from caliper measurements of two orthogonal diameters (x and y, larger and smaller diameters, respectively) by using the formula, volume = xy^2^/2. When the ADR effect on tumor growth delay reached statistical significance between normoglycemic and diabetic groups, tumors were harvested and RNA extracted for analysis of PUMA and DRAM expression by RT-PCR.

### Statistical analysis

Each experiment, unless differently specified, was performed at least three times. Results are expressed as values of mean ± S.D. Statistical significance was determined using Student’s *t*-tests for two sample comparisons and one-way ANOVA analysis for three or more sample comparisons.

## Results

### High glucose (HG) switched the adriamycin (ADR)-induced p53 transcriptional activity from PUMA to DRAM

**Fig. 1:**
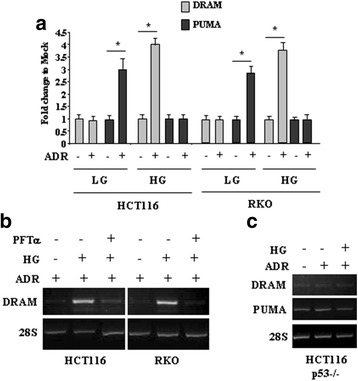
High glucose (HG) switched the adriamycin (ADR)-induced p53 transcriptional activity from PUMA to DRAM. (**a**) RKO and HCT116 cells were kept in low glucose (LG) or high glucose (HG) medium for 24 h and then treated with ADR (2 μg/ml) for 16 h before being assayed for semi-quantitative RT-PCR analysis of PUMA and DRAM gene expression. 28S was used as a control for efficiency of RNA extraction and transcription. Histograms representing quantification of PUMA or DRAM/28S ratio as assessed by densitometric analysis are shown. Densitometric values were quantified using the ImageJ software and normalized to control. The values of control were set to 1. The data are presented as means ± S.D. of three independent experiments. *P < 0.001. (**b**) RKO and HCT116 cells were treated with ADR (2 μg/ml) for 16 h in LG and HG medium, with or without p53 inhibitor pifithrin-α (PFT-α) (30 μM) before being assayed for semi-quantitative RT-PCR analysis of PUMA and DRAM gene expression. 28S was used as a control for efficiency of RNA extraction and transcription. One representative experiment is shown. (**c**) HCT116-p53^−/−^ cells were treated and assayed as in (**a**)

We first evaluated whether the glucose amount influenced the p53 transcriptional activity in response to drug. To this aim, HCT116 and RKO cells were cultured in low glucose (LG) and high glucose (HG) media and treated with adriamycin (ADR). The results of RT-PCR analyses of mRNA levels show that PUMA was greatly induced by ADR in LG, as expected [25], while was not induced in HG (Fig. 1a); on the contrary and opposite to PUMA, DRAM was specifically induced by ADR in HG while was not induced in LG (Fig. 1a). To assess if DRAM was specifically induced by p53 in this setting, as it can be also activated by p73 a p53 family protein [34], we inhibited p53 transcriptional activity with pifithrin-α (PFT-α) [30]. We found that the expression of DRAM, induced by ADR in HG, was efficiently impaired by PFT-α co-treatment (Fig. 1b). Similarly, neither DRAM nor PUMA were induced in HCT116-p53^−/−^ treated with ADR in, respectively, LG and HG (Fig. 1c), demonstrating that both genes were induced by p53 in different glucose culture condition.

### HG culture condition increased autophagy in ADR-treated cells in part depending on p53 activity

**Fig. 2:**
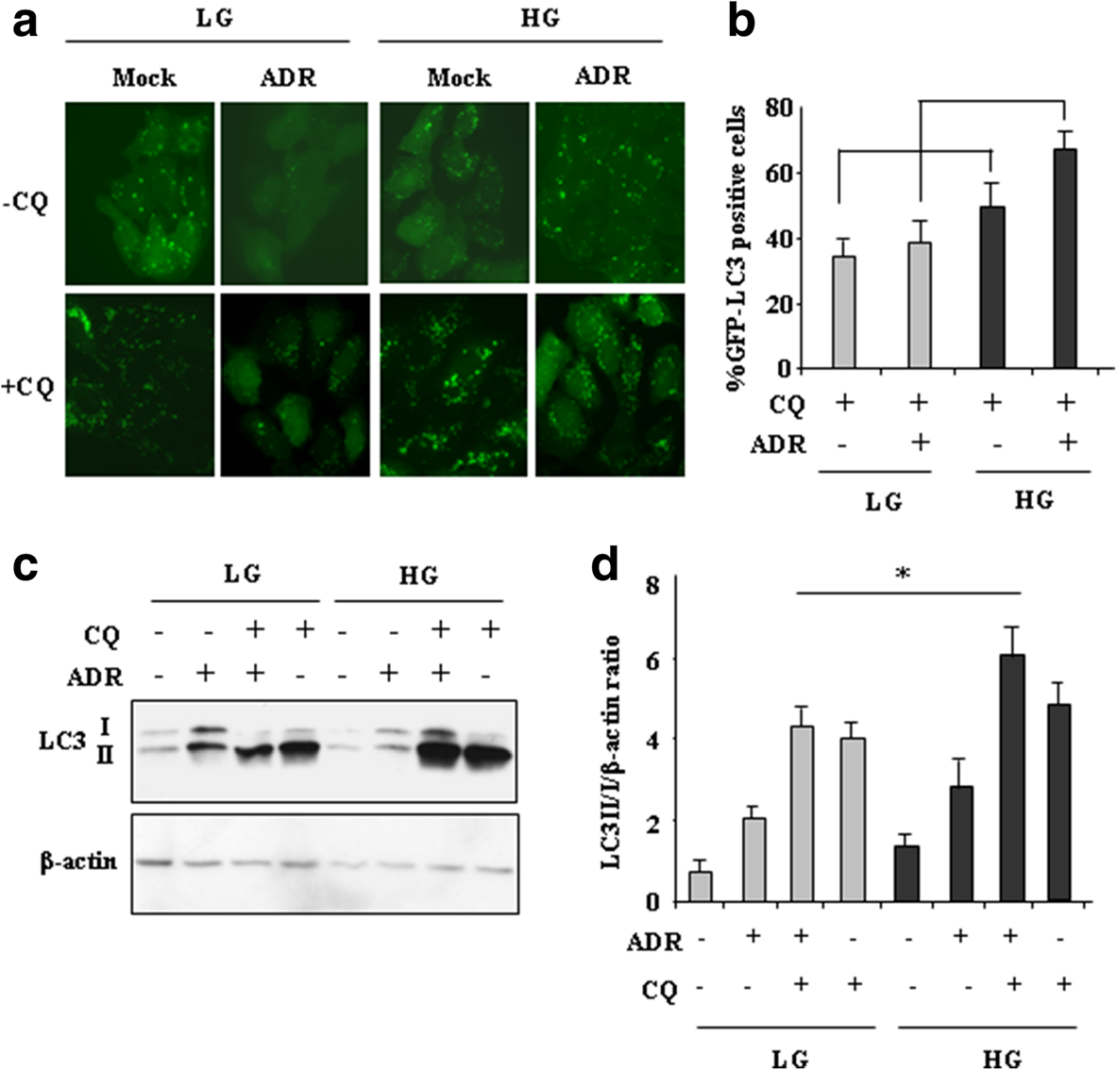
HG increased autophagy in ADR-treated cells. (**a**) RKO stably transfected with GFP-LC3 plasmid were kept in low glucose (LG) or high glucose (HG) medium for 24 h and then treated with ADR (2 μg/ml) for 16, in the presence or absence of autophagy inhibitor chloroquine (CQ, 25 μM) for 4 h before observation to count GFP-LC3 puncta under fluorescence microscopy. Green indicates GFP-LC3. One of 10 representative micrographs is shown. (**b**) The relative number of GFP-LC3-positive cells was calculated from 10 random fields. The data are presented as the means ± S.D. from three independent experiments. (**c**) RKO cells were kept in low glucose (LG) or high glucose (HG) medium for 24 h and then treated with ADR (2 μg/ml) for 16 h in the presence or absence of 25 μM chloroquine (CQ), and the expression of LC3-I/II was measured by western blot analysis. One representative experiment is shown. β-actin was used as internal control. (**d**) Findings as in (**c**) were assessed by quantitative analysis of LC3II-I/β-actin protein levels and shown as histograms. The data are presented as the means ± S.D. from three independent experiments. *P < 0.001

**Fig. 3:**
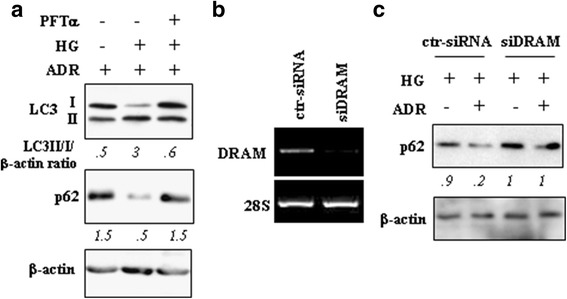
Increase of autophagy of ADR-treated cells in HG was in part depended on p53 activity. (**a**) HCT116 cells were treated with ADR (2 μg/ml) for 16 h in LG and HG medium, with or without p53 inhibitor pifithrin-α (PFT-α) (30 μM) before being assayed for western blot analysis. Densitometric values of LC3II/I/β-actin protein levels were quantified using the ImageJ software and normalized to control. One representative experiment is shown. (**b**) HCT116 cells were transfected with ctr-siRNA and siDRAM and 36 h after transfection DRAM expression was assessed by RT-PCR analysis. One representative experiment is shown. (**c**) HCT116 cells, transfected with ctr-siRNA and siDRAM, were kept in HG medium for 24 h and then treated with ADR (2 μg/ml) for 16 h before the expression of p62 was measured by western blot analysis. Densitometric values of p62/β-actin protein levels were quantified using the ImageJ software and normalized to control and the results are shown under the images. One representative experiment is shown. Anti β-actin was used as protein loading control

To evaluate the occurrence of autophagy, RKO cells were stably transfected with GFP-LC3 plasmid and selected as mixed population. Then, cells were cultured in LG and HG media and treated with ADR. Autophagy induction was evaluated by the appearance of GFP-LC3 puncta, indicating autophagosome formation, The cells displaying more than 5 dots/cell were considered undergoing autophagy, and the measurements were performed in the presence of autophagy inhibitor choroquine (CQ), an inhibitor of the lysosomal function and therefore indicative of autophagic flux, as reported [29, 35]. We found that the LC3 puncta formation in cells cultured in HG was slightly increased compared to cells cultured in LG and that ADR treatment further increased the LC3 puncta formation in HG compared to the same treatment in LG (Fig. 2a, b). Next Western blot analysis of the expression of LC3 (microtubule-associated protein light chain 3) after conversion from LC3-I to its autophagosome membrane-associated lipidated, activated LC3-II form was performed, in the presence and in the absence of CQ. We found that LC3-II conversion, following ADR treatment in LG medium, was greatly increased by HG medium (Fig. 2c, d), suggesting increase of autophagy in HG. We then evaluated whether p53 was involved in autophagy increase. To this aim, p53 transcriptional activity was inhibited by using PFT-α. As shown in Fig. 3a, the LC3-II conversion following ADR treatment in HG medium was impaired by PFTα co-treatment. Analysis of p62, a bona fide autophagic substrate [35], showed that the reduced levels following ADR treatment in HG were counteracted by PFTα co-treatment (Fig. 3a). The role of DRAM was then assessed by using siRNA interference (Fig. 3b). As shown in Fig. 3c, the degradation of p62 in ADR/HG condition was impaired by DRAM interference. Altogether these findings suggest that HG condition increased the ADR-induced autophagy and that such outcome was in part depending on p53 activity and DRAM expression.

### Autophagy inhibition or DRAM silencing restored ADR-mediated cell death in HG

**Fig. 4:**
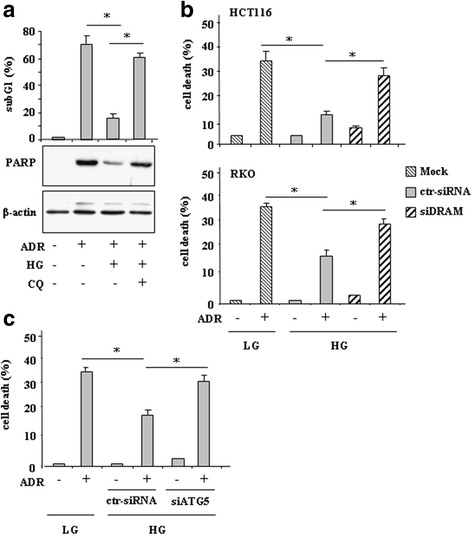
Autophagy inhibition or DRAM silencing restored ADR-induced cell death in HG. (**a**) HCT116 cells were treated with ADR (2 μg/ml for 24 h) in low (−) and high glucose (HG) condition with or without 25 μM CQ (for 16 h). After treatments, cells were in part fixed and stained with propidium iodide (PI) for subG1 evaluation (upper panel) or lysed and analyzed by western immunoblotting to assess PARP cleavage (lower panel); relative quantification of PARP cleavage/β-actin ratio is shown. One representative experiment is shown. Anti-β-actin was used as protein loading control. *P < 0.001. (**b**) RKO and HCT116 cells, transfected with ctr-siRNA and siDRAM or left untransfected, were kept in low glucose (−) or high glucose (HG) medium for 24 h and then treated with ADR (2 μg/ml) for 24 h before the percentage of dead cells was scored by trypan blue exclusion. The data are presented as the means ± S.D. from three independent experiments. *P < 0.001. (**c**) RKO cells, transfected with ctr-siRNA and with siATG5 or left untransfected, were kept in low glucose (−) or high glucose (HG) medium for 24 h and then treated with ADR (2 μg/ml) for 24 h before the percentage of dead cells was scored by trypan blue exclusion. The data are presented as the means ± S.D. from three independent experiments. *P < 0.001

Next, we evaluated whether autophagy was involved in reduction of drug-induced cell death in HG. To this purpose cells were treated with ADR in LG and HG, in the presence or absence of autophagy inhibitor CQ. The results show that the ADR-induced cell death in LG, as assessed by PI staining and FACS analysis, was significantly reduced in HG condition; interestingly, blocking autophagy with CQ rescued the ADR-induced cell death in HG approximately to the levels obtained in LG (Fig. 4a **, upper panel**). CQ alone did not induce cell death (data not shown). In agreement, Western blot analysis show that the expression of the apoptosis marker (cleaved) PARP in response to ADR in LG was reduced by HG and rescued by CQ co-treatment (Fig. 4a **, lower panel**). To further evaluate the role of autophagy, depletion of ATG5, one of the members of the ATG family necessary for autophagy because of its role in autophagosome elongation [36], was performed with specific siRNA. We found that ATG5 knockdown rescued the cell death inhibited in ADR/HG condition to almost the levels obtained by ADR in LG (Fig. 4b). Next the role of DRAM was assessed in both RKO and HCT116 cells. The results show that the ADR-induced cell death in LG and significantly reduced by ADR treatment in HG condition, was mostly restored by silencing DRAM with siRNA (Fig. 4c). Altogether, these data suggest that autophagy and/or DRAM expression were responsible, at least in part, of reduced cancer cell death in response to ADR in HG condition.

### Autophagy inhibition or DRAM silencing restored ADR-mediated PUMA transcription in HG

**Fig. 5:**
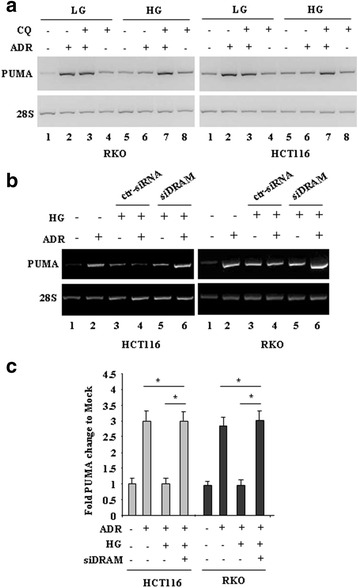
Autophagy inhibition or DRAM silencing restored ADR-induced PUMA transcription in HG. (**a**) RKO and HCT116 cells were kept in low glucose (LG) or high glucose (HG) medium for 24 h and then treated with ADR (2 μg/ml) for 16 h in the presence or absence of 25 μM CQ, before being assayed for semi-quantitative RT-PCR analysis of PUMA gene expression. 28S was used as a control for efficiency of RNA extraction and transcription. One representative experiment is shown. (**b**) RKO and HCT116 cells, transfected with with siRNA and siDRAM or untransfected, were kept in low glucose (−) or high glucose (HG) medium for 24 h and then treated with ADR (2 μg/ml) for 16 h before being assayed as in (**a**). One representative experiment is shown. (**c**) Densitometric values of three independent experiments as in (**b**) were quantified using the ImageJ software and normalized to control. The values of control were set to 1. The data are presented as fold of induction ± S.D. *P < 0.005

Next we evaluated whether blocking autophagy and/or DRAM could influence PUMA transcription in HG. RT-PCR analyses of mRNA levels in RKO and HCT116 cells show that PUMA expression was induced by ADR in LG while was not induced in HG (Fig. 5a **, compare lane 2 with lane 6**), as expected; interestingly, PUMA was induced by ADR in HG only in the presence of CQ (Fig. 5a **, compare lane 6 with lane 7**), while the ADR-induced PUMA expression in LG was not further increased by CQ co-treatment (Fig. 5a **, compare lane 2 with lane 3**). As opposite to PUMA, DRAM expression was induced by ADR only in HG, as seen above, but inhibited by CQ co-treatment (data not shown). Interestingly, PUMA was induced by ADR in HG following siRNA silencing of DRAM (Fig. 5b **, compare lane 4 with lane 6**), as assessed by densitometric analysis (Fig. 5c). Altogether, these findings suggest that the p53 pro-apoptotic function, impaired in HG, could be restored by autophagy inhibition or DRAM depletion. Therefore, we hypothesize that, downstream of p53, autophagy might sustain inhibition of p53 pro-apoptotic function in a regulatory loop.

### Diabetes reduced the effect of chemotherapy that correlated with increased DRAM and reduced PUMA gene expression, in vivo

**Fig. 6:**
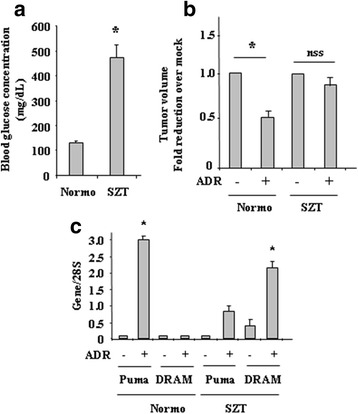
Diabetes reduced the effect of chemotherapy that correlated with increased DRAM and reduced PUMA gene expression, in vivo. (**a**) Blood glucose concentration was evaluated in normoglycemic (Normo) and streptozotocin (SZT)-treated mice (diabetic). Data are presented as means ± S.D. *P < 0.005. (**b**) After mice reached a glucose concentration exceeding 300 mg/dl (considered diabetic) upon SZT treatment, solid tumors were obtained by injecting i.m. RKO cells on the flank of each mouse. ADR treatment was performed when the tumors became palpable. Ten days after ADR treatment, the size of tumors showed statistical significant growth delay in normoglycemic versus SZT mice. The data are presented as fold reduction ± S.D. *P < 0.005. (**c**) Tumors measured in (**b**) were then explanted from normoglycemic (Normo) and streptozotocin (SZT)-treated mice and total mRNA was analysed by RT-PCR of PUMA and DRAM gene expression. 28S was used as a control for efficiency of RNA extraction and transcription. (**c**) Densitometric analysis of gene expression in (**b**) was plotted as expression ratio to 28S. *P < 0.001

Finally, the biological effect of the hyperglicemic microenvironment on drug response was evaluated in a nude mouse model of Streptozotocin (STZ)-induced diabetes [33], to recapitulate the in vitro results. After the mice achieved a consistent diabetic state, as evaluated by blood glucose concentration exceeding 300 mg/dL, compared to the normal group that maintained blood glucose concentration around 100 mg/dL (Fig. 6a), we generated tumor xenografts by injecting RKO cells, as previously reported [37, 38], in both diabetic and normoglycemic groups. Cells were implanted into nude mice by i.m. injection and allowed to develop into palpable tumor nodules (about 300 mm^3^) at the injection sites. The mice were then treated with ADR and tumor volumes were calculated from caliper measurements. Ten days after injection only normoglycemic mice treated with ADR displayed significant tumor growth delay (ADR versus Mock: *P < 0.001), compared to the same treatment in diabetic (SZT) mice (Fig. 6b). Then, tumors were harvested and mRNA extracted for p53 target gene expression by RT-PCR analyses. The results show that PUMA was significantly induced by ADR treatment in normoglycemic mice (Fig. 6c) while weakly induced in SZT mice; on the contrary, DRAM was induced by ADR treatment in diabetic mice while was not induced in normoglycemic ones, in agreement with the above in vitro data. This experiment recapitulated the in vitro results and underlined how the diabetic condition might negatively influence tumor response to drugs by impairing p53 pro-apoptotic activity.

## Discussion

PUMA is a potent inducer of apoptosis, whereas inhibiting PUMA curbs apoptosis associated with tissue injury thus inducing therapeutic resistance [10, 11]. DRAM, by itself or in conjunction with other factors downstream of p53, can induce autophagy. Intriguingly, DRAM is also involved in p53-induced cell death, although DRAM has no detrimental effect on cell viability when expressed alone [18, 19]. PUMA and DRAM are two p53 target genes and it is well known that p53 after its activation can select the final biological outcome depending on the triggered transcriptional program [1, 2]. Indeed, although p53 is better known for its proapoptotic activities, it can also induce prosurvival effects, particularly under mild stress conditions. Starting from our previous finding that HG reduces p53 apoptotic activity [25, 26], we found here that the HG condition changed the p53 response upon ADR treatment, driving p53 transcriptional activity from PUMA (death) to DRAM (autophagy) mutual antagonism and that such interplay correlated with impaired drug-induced cell death. Despite DRAM has been shown to induce also autophagic cell death, in our system it played a pro-survival role since silencing DRAM restored drug-induced cell death inhibited by HG condition. We found that DRAM expression increased the HG-induced autophagy that, in turn, inhibited apoptosis and facilitated the resistance of tumor cells to anticancer agents, in agreement with some studies [22, 23]. Further studies will elucidate whether the PUMA to DRAM differential expression is evident also in tumor tissues and whether it might be linked to tumor resistance to drugs.

The PUMA to DRAM interplay was the result of p53 activation since blocking p53 transcriptional activity or using p53 null cells abolished such expression. Given that p53 can induce apoptosis in response to high genotoxic stress, it is plausible that HG might modify the way through which p53 selectively induced gene transcription. We know that p53 phosphorylation at serine 46 (Ser46) is a critical modification for induction of irreversible apoptosis and that inhibition of such phosphorylation by HG contributes to chemoresistance and reduction of p53-induced apoptosis [39, 40]. We also know that mainly HIPK2 phosphorylates p53Ser46 and that HG triggers HIPK2 degradation, impairing p53 apoptotic response [25, 41]. In agreement, we found that PUMA expression is related to p53Ser46 modification [25, 26]. However, although we found here mutual interplay between PUMA to DRAM transcription, whether p53-induced DRAM expression depends on specific p53 modification (i.e., phosphorylation, acetylation, ect.) and/or different extent of genotoxic stress, needs to be clarified.

DRAM is a lysosomal protein and a stress-induced regulator of autophagy that was firstly discovered by microarray analysis of mRNA species responsive to p53 and associated to autophagy induction [18], although the effect of DRAM-mediated autophagy has not been clarified until now. Starvation has been shown to activate the PI3K/AKT pathway that inhibits apoptosis by DRAM-mediated autophagy in hepatocellular cancer cells [42]. We can speculate that the loss of HIPK2/p53Ser46 phosphorylation in high glucose changes the p53 transcriptional activation from pro-apoptotic (PUMA) gene toward autophagy (DRAM) gene. Thus, DRAM silencing restored the ADR-induced cell death in high glucose and inhibited autophagy, highlighting a pro-survival effect of DRAM, likely related to autophagy induction. Whether this effect is cell specific or context–dependent needs further studies.

Autophagy has been shown to have a pro-survival effect in response to chemotherapy when for instance is activated by endoplasmic reticulum stress or by reactive-species oxygen (ROS) and to induce chemoresistance [23, 43–46]. Our present studies show that blocking autophagy with CQ or by depletion of ATG5 gene, restored the ADR-dependent cell death, highlighting a pro-survival effect of autophagy in this model. Interestingly, blocking autophagy restored PUMA transcription. This was somehow surprisingly. Preliminary findings indicate that HIPK2 might be involved in PUMA to DRAM interplay (data not shown). Further studies will be needed to address whether HIPK2 might be degraded also by autophagy other than by proteasome, as previously reported [47].

## Conclusions

In conclusion, these findings uncover the antagonistic role of PUMA and DRAM to govern drug-induced cell death, highlighting how metabolic conditions such as hyperglicemia might change p53 response to anticancer therapies. These findings might be exploited to design personalized therapies that combine the use of autophagy inhibitors to improve the efficacy of chemotherapy in hyperglicemic patients.

## Abbreviations

ADR: Adriamycin
CQ: Chloroquine
DMEM: Dulbecco’s Modified Eagle’s medium
DRAM: Damage-regulated autophagy modulator
FACS: Fluorescence Activated Cell Sorting
FBS: Fetal bovine serum
HBSS: Hanks’ Balanced Salt solution
HG: High glucose
LC3: Microtubule-associated protein light chain 3
LG: Low glucose
PI: Propidium iodide
pifithrin-α: PFT-α
PUMA: p53 upregulated modulator of apoptosis
PVDF: polyvinylidene difluoride
RT-PCR: reverse transcription polymerase chain reaction
SD: Standard deviation
siRNA: small-interference RNA
STZ: Streptozotocin
